# Characterization and Transferring of Human Rotavirus Double-Layered Particles in MA104 Cells

**DOI:** 10.5812/jjm.10375

**Published:** 2014-06-01

**Authors:** Ali Teimoori, Hoorieh Soleimanjahi, Manoochehr Makvandi

**Affiliations:** 1 Department of Virology, Faculty of Medical Sciences, Tarbiat Modares University, Tehran, IR Iran; 2 Department of Virology, Ahvaz Jundishapur University of Medical Sciences, Ahvaz, IR Iran

**Keywords:** Rotavirus, Transfection, Viral Plaque Assay

## Abstract

**Background:**

Rotavirus (RV) is a major cause of gastroenteritis in infants and children and is one of the most severe public health problems. Rotaviruses outer layer contains two proteins including VP4 and VP7. These proteins are necessary for host-cell binding and penetration. TLP (triple layer virus particle) of RV is a complete infectious virion that binds to the target cells and internalized at the cytoplasm. The DLP (double layer virus particle) is a non-infectious particle that is formed through exclusion of the outer layer proteins including VP4 and VP7. These DLPs are the transcriptionally active forms of rotavirus.

**Objectives:**

The aim of this study was to transfer DLP of RV into cytoplasm of MA104 cells by Lipofectamine and to analyze their replication.

**Materials and Methods:**

Initially, rotavirus was purified by CsCl discontinuous gradient and DLP was separated from TLP based on density differences. For confirmation, sodium dodecyl sulfate polyacrylamide gel electrophoresis (SDS-PAGE) of the proteins were conducted Then the purified DLP of RV was transferred into MA104 cells using Lipofectamine.

**Results:**

We attempt to avoid the attachment and entry of the rotavirus by using Lipofectamine to mediate the delivery of viral particles directly into the cytoplasm. DLP was endocytosed into the cytoplasm following treatment by Lipofectamine and then replicated in cytoplasm.

**Conclusions:**

Therefore the non-infectious DLPs were became infectious if introduced into the cytoplasm of permissive and cancerous cells, without passing attachment and entry process.

## 1. Background

Rotavirus (RV) is a major cause of gastroenteritis in infants and children and is one of the most severe public health problems, worldwide especially in developing countries of Africa and Asia continents (1). Two rotavirus vaccines, which are licensed in the United States are RotaTeq^®^ and Rotarix^®^. These rotavirus vaccines are safe and effective to prevent severe diarrhea (2). Rotaviruses have a segmented genome packaged within a triple layer virus particle (TLP). The outer layer of the virion contains two proteins including VP4 and VP7 (3). These proteins are molecular machinery for host-cell binding and penetration. VP4 and VP7 are a set of spike-like projections and a shell, respectively. The intermediate layer of rotavirus capsid composed of 260 trimer of VP6 proteins and the inner layer is composed of 120 molecules of VP2 (4). 

Several molecules including gangliosides GM1, GM3, integrins and heat shock cognate protein 70 have been involved as attachment receptors for rotaviruses (5, 6). In cell culture, rotavirus showed two forms of triple layer and double layer particles (DLP). TLP of RV is complete infectious virion and binds to target cells, which internalized at the cytoplasm. The DLP is a noninfectious particle which forms through removal of the outer layer proteins (VP4 and VP7). These DLPs are transcriptionally active forms of rotavirus and capable to produce virus within the target cells (7). Since non-treated DLPs cannot internalize to the target cells. In this study, DLP of human rotavirus RV4 purified with CsCl and transfected with Lipofectamine®. For confirmation of virus biological activity and virus production the plaque assay was done. By the use of Lipofectamine, Transferring of non-infectious DLPs in target cells mimic native rotavirus infection and may be used for the treatment of cancerous cells.

## 2. Objectives

In this report we showed that the DLP particles are transferred by treating with transfectant reagents such as Lipofectamine, which they can be internalized into several kinds of nonpermissive mammalian cells as well as cancerous cells for oncolytic purpose.

## 3. Materials and Methods

### 3.1. Virus and Cells

MA104 cells (Pasture, Iran) were cultured in 175-cm^2^-flask containing DMEM (DMEM, Gibco) supplemented with 10% fetal bovine serum (FBS) at 37°C and 5% CO2. Confluent Monolayers of MA104 cells were infected with human rotavirus RV4 at a multiplicity of infection (MOI) of 0.05. For virus activation, trypsin (porcine pancreatic type IX: Sigma) at a final concentration of 10 μg/mL was added and incubated for 1 hour at 37° C (8). The infected cells were lysed through three freezing-thawing cycles in order to release cell associated virus.

### 3.2. DLP Purification

Discontinous isopycnic density-gradient centrifugation was established for purification of rotavirus. Two forms of RV particles have been observed using CsCl gradient (8, 9). The density of TLP and DLP were ∼1.36 g/cm^3^ and ∼1.38 g/cm^3^, respectively. Two hundred milliliters of rotavirus cell lysate centrifuged at 110000 × g, at 4°C for 1.5 hours using an SW28 rotor in order to precipitate the virus particles and cellular debris, then the pellet resuspended in 40 mL of Tris sodium chloride (TNC) buffer (20 mM, Tris.Cl, 100 mM NaCl, 1 mM CaCl2). Resuspended solution were mixed with an equal volume of Freon (Trichlorotrifluoroethane) and centrifuged at 4100 × g at 4°C for 10 minutes. Upper phase of solution transferred into tubes and centrifuged at 110000 × g, at 4°C for 1.5 hours using an SW28 rotor to pellet virus particles. Virus pellet suspended in 6 mL of TNC buffer. The discontinuous gradient density of 1.4 g/cm3 CsCl for lower phase and 1.2 g/cm3 CsCl for upper phase was prepared and virus suspension poured and centrifuged at 110000 × g, at 4°C for 3 hours using a 50-Ti rotor. The two cloudy bands have been shown. Upper and lower bands are infectious triple-layered and non-infectious DLPs, respectively. The DLP band was isolated and then the CsCl was eliminated by dialyzing against TNC buffer containing 0.5 M Ethylene diamine tetraacetic acid (EDTA), overnight (8).

### 3.3. Sodium Dodecyl Sulfate Polyacrylamide Gel Electrophoresis (SDS-PAGE) of Double Layer Particle (DLP) and Triple Layer Particle (TLP)

The purified DLP and TLP of viruses were resuspended individually with 5X sample buffer (Fermentas). The samples were immediately denatured prior to use by incubating at 95°C for 5 minutes in the presence of β-mercaptoethanol. Proteins were resolved on 12% SDS-PAGE (Laemmli system) and visualized by Coomassie Blue R-25 0.1% stain.

### 3.4. Triple Layer Particle (DLP) Transfection

The purified DLP was mixed with Lipofectamine and transfected into MA104 cells. As a control of experiment, DLP without Lipofectamine was serially diluted (10^-1^ to 10^-6^) and transfected into cells using the same protocol. To minimalize the toxicity to the cells and efficient lipofection of viral particles, the amount of Lipofectamine was optimized based on the manual of Lipofectamine 2000 Reagent. Three concentrations of the transfectant with DLP of RV were applied (Table 1).

**Table 1.** Transfection of DLP of Rotavirus by Lipofectamine Into MA104 Cells ^a^

-	Component	6 Well Plate for Test	6 Well Plate for Control of Trasfection
**1**	DMEM (High glucose)	890 uL	890 uL
**2**	Lipofectamine 2000 Reagent	4, 6, 10 uL	-
**3**	Number of cells	5×10^5^	5×10^5^
**4**	DLP purified	100 uL	100 uL
**5**	Mixed 1, 2 and 4, Incubated for 5 minutes at room temperature		
**6**	Mixed component tenfold diluted (10^-1^ to 10^-6^) into MA104 confluent cells incubated at 37°C		

^a^ The plates were incubated for 1 hour at 37° C to transfect the virus.

### 3.5. Plaque Assay

The plaque assay is one of the common biological assays using for the quantification of RV. This assay is based on the CPE, which was caused by active and replicapable forms of RV in cultured cells and introduced to plaque-forming units per milliliter of virus (PFU/mL). Neutral red was used as a vital stain for visualization of the plaques. The living cells uptake the neutral red, whereas the lysed infected cells are transparent and form the plaque (without uptake of neutral red) (10, 11) After transfection the agar containing 1:1 mixture of 1.6% (w/v) agar and serum-free 2× DMEM was overlaid. Once the agar was solidified, the plates were transferred to a 37◦C-incubator until plaques are visible. The same procedure was applied for control of experiment.

## 4. Results

### 4.1. Propagation of Rotavirus in MA104 Cells

**Figure 1.**
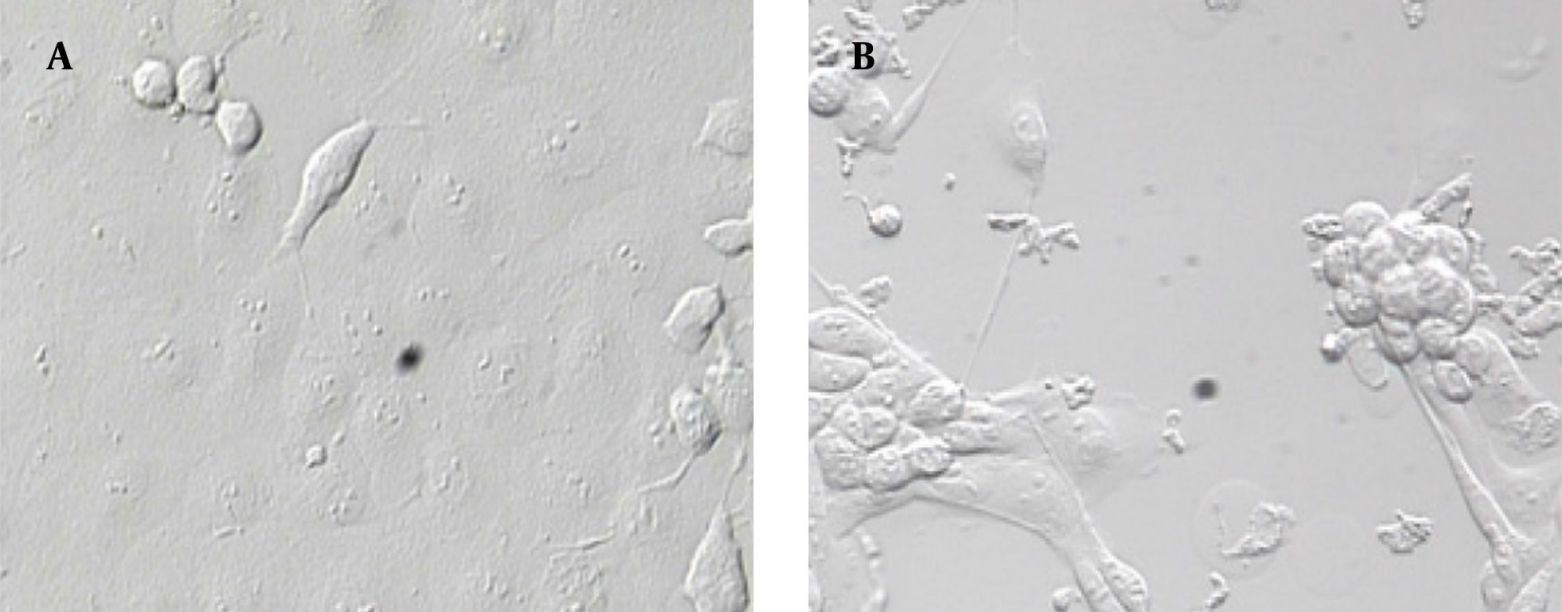
Infected and Non Infected MA104 Cells A: confluent monolayer noninfected MA104 cells; B: infected MA104 cells and CPE.

For RV propagation, infections are commonly performed at low MOI (0.05) to reduce the generation of mutations, gene rearrangements and to optimal yields of infectious virus. CPE has progressed to fully disrupt, three days after infection with human rotavirus RV4 (Figure 1). Rotavirus was released by the lysis of MA104 infected cells and total cell lysate and medium was collected.

### **4.2. Double Layer Particle **(**DLP**)** Purification of Human Rotavirus**

**Figure 2.**
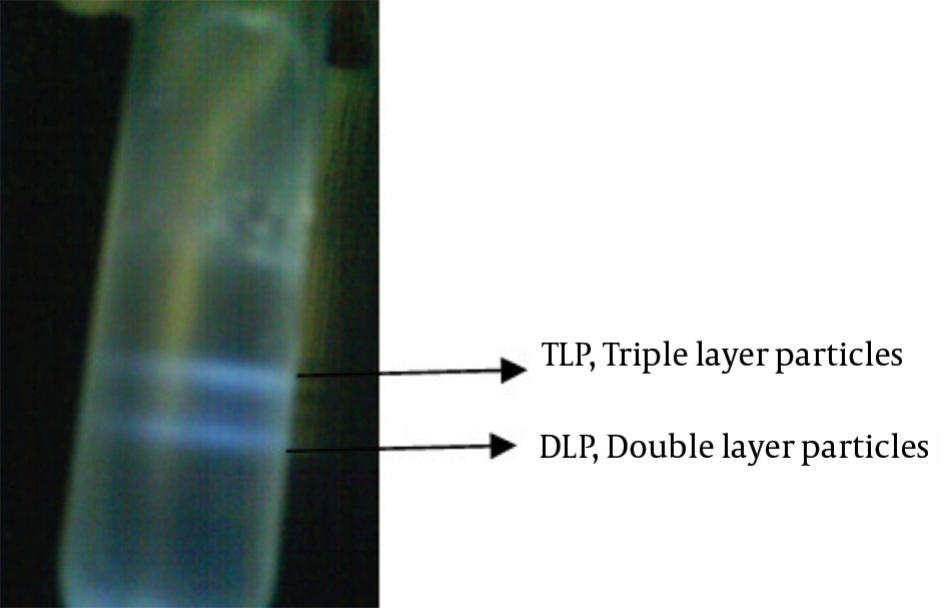
Two Distinct Bands of RV Particles Using a CsCl Gradient The upper band compromises of infectious, triple-layered particles (TLPs; density 1.36 g/cm^3^) and the lower one compromises of noninfectious, double-layered particles (DLPs; density 1.38 g/cm^3^).

Centrifuged tube was contained two cloudy, whitish-colored bands which was visible in darkened room with an inverted light source. The upper phase of band was discarded by a pipet for collection of TLP and DLP bands separately in two individual tubes (Figure 2). To remove the probable trace of TLP, 10 mM EDTA was added to the dialysis buffer.

### 4.3. Protein Content Analysis of TLP & DLP by SDS-PAGE

**Figure 3.**
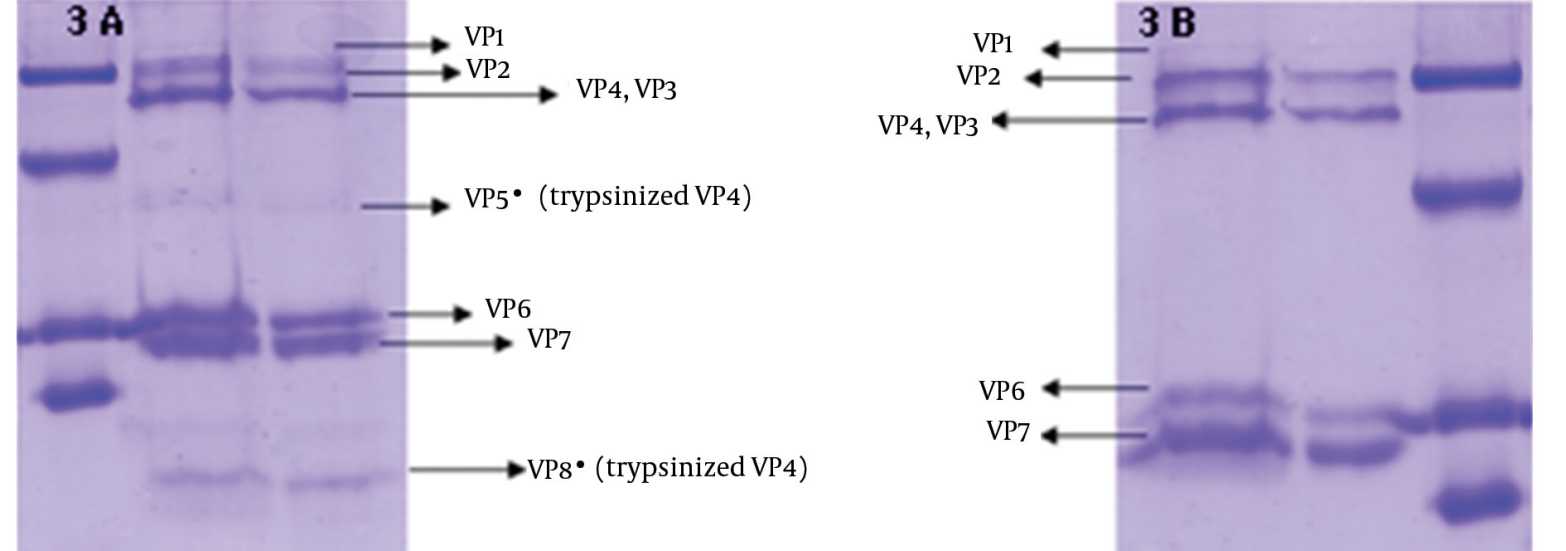
Electrophoretic Pattern of Triple Layer Particle & Double Layer Particle A: TLP of trypsinized human rotavirus particle in which the VP4 protein was cleaved to VP5* and VP8*; B: TLP of not trypsinized human rotavirus with an intactVP4.

**Figure 4.**
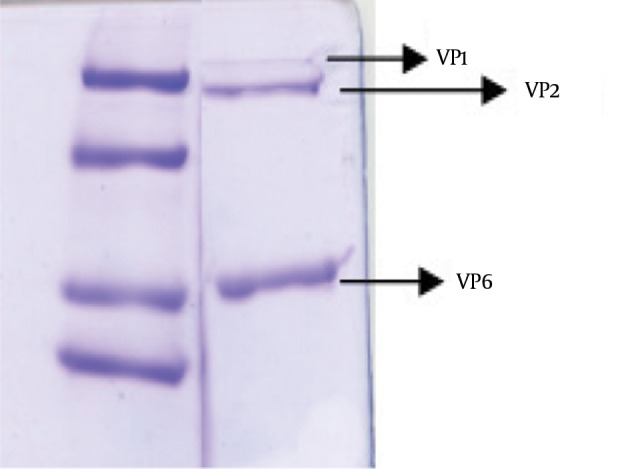
Electrophoretic Pattern of Human Rotavirus DLP DLP of human rotavirus containing of four proteins including VP1, 2, 3 and VP6, traces mounts of VP3 and not detected by Coomassie Blue staining.

To verify the absence of outer capsid proteins in DLP, the treated band was analyzed by SDS PAGE. In addition TLP was determined as a control of outer capsid protein. Outer capsid proteins consist of VP7 (MW: 38 kDa) and VP4 (MW: 88 kDa). In this study, the analysis of the TLP polypeptides pattern revealed the expected pattern of trypsin-treated rotavirus with cleavage of VP4 to yield VP5 (MW: 60 kDa) and VP8*(MW: 28 kDa) (12). Figure 3 was compatible with other researches' findings. The DLP is composed of the remaining four structural proteins including VP1 (MW: 125 kDa), VP2 (MW: 102 kDa), VP3 (MW: 88 kDa) and VP6 (MW: 44) (7, 13), is shown in Figure 4.

### 4.4. Plaque Assay as a Biological Method for Virus Replication

**Figure 5.**
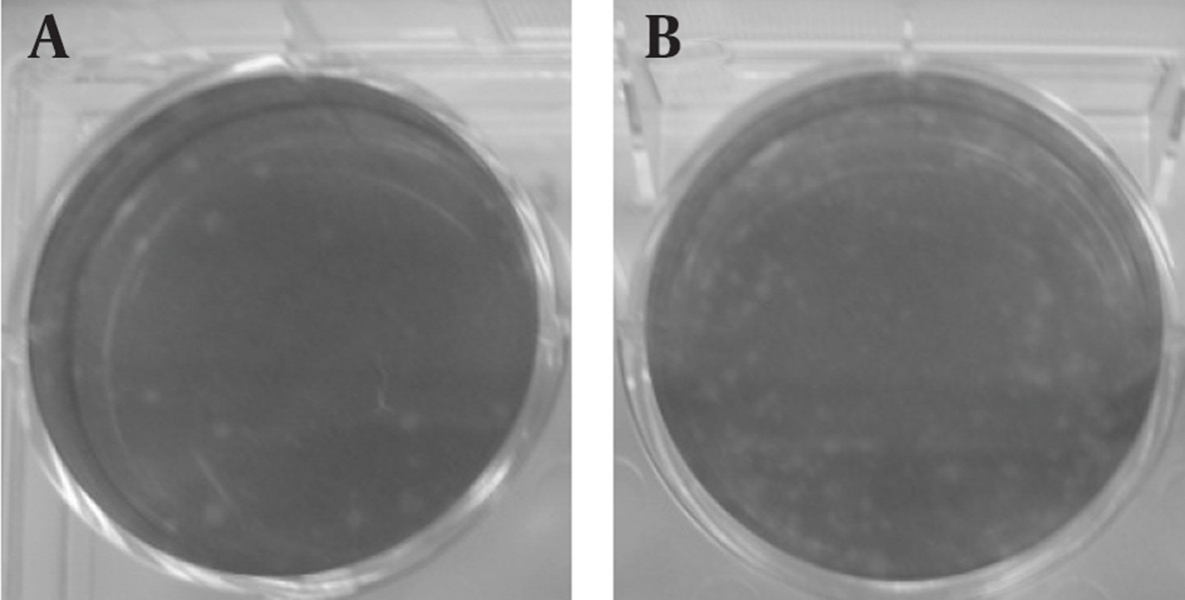
Results of Plaque Assay Using the DLP of Rotavirus Showing the Transfer of the Particles Into Permesive MA104 Cells At 96 hours of post-infection, the cells were stained with neutral red. A: tenfold dilution (10^-4^) of DLP treat with transfectant; B: 10^-3^ of DLP treats with transfectant.

**Figure 6.**
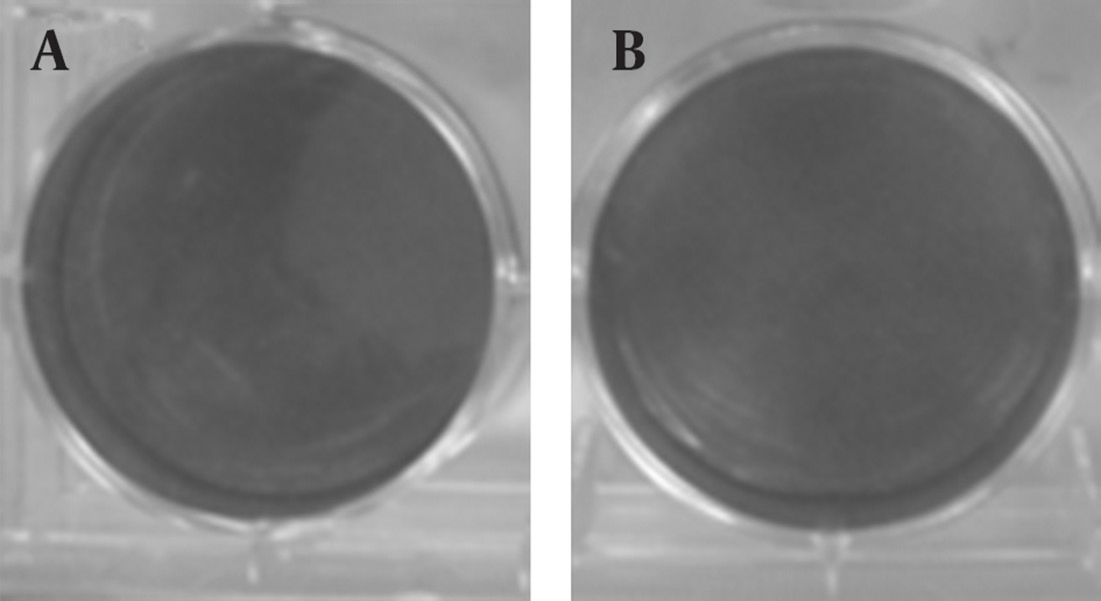
Plaque Assay by DLP Without Transfectant A: Tenfold dilution (10^-4^) of DLP without transfectant; B: 10^-3^ DLP without transfectant.

The plaque assay is the most elegant, quantitative, and useful biological assay for viruses. Four to five days after DLP inoculation, plaques are visible (Figure 5). As control, DLP without transfectant was inoculated and plaque was not visible (Figure 6). Taken together, transferring of DLP into the cytoplasm resulted in production of virus progenies and replication of virus resumed.

## 5. Discussion

Rotaviruses are non-enveloped viruses that infect enterocytes in small-intestinal and entirely replicate in the cell cytoplasm (1, 14). Rotavirus particles consist of three concentric layers of protein or triple layered particles (TLP), which is composed of two proteins, VP4 and VP7, in its outer layer. The rotavirus virion attaches to the target cell receptor through VP8* (produced by the cleavage of VP4 into VP5* and VP8*). The mechanism of rotaviruses entry to cells remains controversial and different pathway for virus entry has been presented in several published researches (15, 16). Calcium-dependent, clathrin-coated, caveolae-mediated endocytosis and direct penetration are suggested methods of virus entry, but further detailed studies are required to understand this pathways (17). However, some of these studies using specific inhibitory drugs have definitely ruled out the classical endocytic mechanism of trypsin-primed rotavirus. 

Despite some contradictory results, the prevailing concept from these studies shows rapid kinetics of internalization enters through direct penetration, leading to a productive infection by enzymatic treatment. Trypsin-activated Rotaviruses was internalized within a few minutes (half-time of 3 to 5 minutes) (18, 19). Following penetration into the target cells, the outer capsid is loosed and the double-layered particle (DLP) released into the cytosol. Subsequently the internal polymerase complex activates (VP1 and VP3) to transcribe capped positive-sense RNA ((+) RNAs) from each of the 11 double-stranded RNA (dsRNA) segments (20, 21).

In this experiment, rotavirus was purified by CsCl discontinuous gradient. Buoyant density in CsCl gradient of rotavirus DLP and TLP is different, two kinds of particles were separated and purified. Since DLPs are heavier and denser than TLPs, they move faster than TLPs in CsCl gradient. The DLP are non-infectious and disable to attach and entre, but these particles can be active and replicapable in the cytoplasm. Polycations as well as Lipofectamine have been shown to increase the infectivity of several viruses and refinement of virus entry (22, 23).

In this study, we attempt to bypass the attachment and entry of the rotavirus by using Lipofectamine to mediate the delivery of viral particles directly into the cytoplasm. Probably the DLPs enter into the cytoplasm following treatment by Lipofectamine. Plaque assay is used for confirmation of virus replication, production and biological activity. Based on the results, the treatment of DLPs with Lipofectamine does not have influence on virus replication. For verification of DLP purity, SDS-PAGE was applied and VP6, VP1, 2 proteins have been detected. In DLP, VP6 is the major constituent with 260 trimers of VP6 protein. As shown in previous experiments, the transfer of DLP in non-permissive cell lines could mediate infection (3) and perhaps this method can be used for the transfer of rotavirus DLP in non-permissive and cancerous cells for oncolytic purposes.
